# Co-infection of ST2_IP_ carbapenem-resistant *Acinetobacter baumannii* with SARS-CoV-2 in the patients admitted to a Tehran tertiary referral hospital

**DOI:** 10.1186/s12879-021-06642-2

**Published:** 2021-09-08

**Authors:** Alireza Abdollahi, Amir Aliramezani, Mohammadreza Salehi, Mahsa Norouzi Shadehi, Sedighe Ghourchian, Masoumeh Douraghi

**Affiliations:** 1 Department of Pathology, School of Medicine, Imam Khomeini Hospital Complex, Tehran University of Medical Sciences, Tehran, Iran; 2 Department of Infectious Disease, School of Medicine, Imam Khomeini Hospital Complex, Tehran University of Medical Sciences, Tehran, Iran; 3 Division of Microbiology, Department of Pathobiology, School of Public Health, Tehran University of Medical Sciences, PO Box: 14155-6446, Tehran, Iran

**Keywords:** Carbapenem-resistant *Acinetobacter baumannii* (CRAB), COVID-19, *oxa23*, *oxa24*, *oxa58*, SARS-CoV-2, Sequence type

## Abstract

**Background:**

Carbapenem-resistant *Acinetobacter baumannii* (CRAB) is among the most concerning cause of healthcare-associated infections (HAI) due to its high level of antibiotic resistance and high mortality. In the era of the COVID-19 pandemic, the key priority of infection control committees is to contain the dissemination of antibiotic resistant Gram-negative bacteria. Here, we aimed to timely recognize the emergence of CRAB in COVID-19 cases admitted to the wards of a tertiary referral hospital and to identify the genetic relatedness of the isolates.

**Methods:**

From 30 March to 30 May 2020, a total of 242 clinical samples from COVID-19 cases were screened for CRAB isolates using standard microbiologic and antibiotic susceptibility tests. The PCRs targeting *oxa23*, *oxa24*, *oxa58*, *bla*_TEM_ and *bla*_NDM-1_ genes were performed. Two multiplex PCRs for identifying the global clones (GC) of *A. baumannii* were also performed. The sequence type of CRABs was determined using Institut Pasteur (IP) multilocus sequence typing (MLST) scheme.

**Results:**

Eighteen CRAB isolates were recovered from COVID-19 patients with the mean age of 63.94 ± 13.8 years. All but 4 COVID-19 patients co-infected with CRAB were suffering from an underlying disease. Death was recorded as the outcome in ICUs for 9 (50%) COVID-19 patients co-infected with CRAB. The CRAB isolates belong to GC2 and ST2_IP_ and carried the *oxa23* carbapenem resistance gene.

**Conclusion:**

This study demonstrated the co-infection of CRAB isolates and SARS-CoV-2 in the patients admitted to different ICUs at a referral hospital in Tehran. The CRAB isolates were found to belong to ST2_IP_, share the *oxa23* gene and to have caused several outbreaks in the wards admitting COVID-19 patients.

## Background

*Acinetobacter baumannii* is an emerging Gram-negative pathogen that causes a variety of healthcare-associated infections (HAI), including ventilator-associated pneumonia (VAP), central line-associated bloodstream infections and catheter-associated urinary tract infections [1]. The outbreaks of *A. baumannii* infections tend to occur in intensive care units (ICUs) with critically ill patients [2].

Carbapenems are considered as the first-line antimicrobials for treatment of infections caused by antibiotic-resistant Gram-negative bacteria [3]. The carbapenem resistance emerged in *A. baumannii* shortly after the introduction and therapeutic use of carbapenems and disseminated globally [4, 5]. To date, two major clonal complexes, namely global clones 1 and 2 (GC1 and GC2), have been reported to be mostly responsible for the resistance of this microorganism [6]. The emergence of carbapenem-resistant *A. baumannii* (CRAB) has been a great concern in clinical practice, as it reduces the therapeutic options for patients [7]. The Centre for Disease Control (CDC) listed the multidrug-resistant *Acinetobacter* as “Serious threat” in 2013 [8]; however, it reported CRAB as “Urgent threat” in 2019. This threat level increased due to the lack of new antibiotics for treatment as well as the widespread dissemination of resistance among the isolates in healthcare settings [9]. The carbapenem resistance in *A. baumannii* is significantly associated with the carbapenem hydrolysing oxacillinases encoded by the *oxa23*, *oxa24* and *oxa58* genes which are found on mobile genetic elements and can be disseminated among bacteria [10, 11].

Following the first report of two cases of coronavirus disease of 2019 (COVID-19) in Qom province, Iran, in February 2019, other provinces including the capital city, Tehran, was also affected [12]. With the beginning of the first wave of COVID-19 infection and spreading throughout the country, the referral hospitals in the metropolitan area of Tehran started to provide therapeutic and diagnostic services for COVID-19 patients.

The presence of indwelling or invasive medical devices in severely ill patients receiving critical care, intubation or mechanical ventilation [13] and the length of stay (LOS) in the ICU are potentially associated with an increased risk of contracting HAIs, particularly VAP [13]. Due to the lack of FDA-approved treatment [14], the management of patients with COVID-19 will be highly problematic especially when co-infected with antibiotic-resistant bacteria commonly distributed in hospitals. Therefore, the timely recognition of antibiotic-resistant bacteria is important in order to combat the antibiotic resistance and implement the infection and prevention control (IPC) practices. Here, we aimed to detect the CRAB isolates in the wards with COVID-19 cases and determine the genetic relatedness of the CRAB isolates.

## Methods

### Setting and sampling

Between 30 March and 30 May 2020, a total of 242 clinical samples from COVID-19 patients admitted to one of the five different ICUs at a tertiary referral hospital in Tehran, Iran, were submitted to the laboratory for CRAB screening. This hospital is the largest hospital in Iran with more than 1000 active beds, which serves both admitted and non-admitted patients from Tehran and other cities. Prioritising COVID-19 patients, this hospital housed these patient following the official announcement of the SARS-CoV-2 outbreak in the country. The hospital laboratory began testing specimens obtained from cases suspected of COVID-19 using the reverse transcription-polymerase chain reaction (RT-PCR) kit (Sansure Biotech Inc., China) and targeting SARS-CoV-2. The members of the infection control committee at this hospital strictly adhered to infection control and cross transmission prevention measures.

Patients’ information including the age, gender, ward of admission, source, length of stay in ICU, antibiotic treatment, need for tracheal intubation and/or mechanical ventilation, history of underlying disease and outcome of patients before discharge was obtained from their medical files.

### Isolates and identification

One isolate per COVID-19 patient was included in this study. A single colony of each isolate was identified based on the phenotypic tests. Genomic DNA extraction was performed using the standard phenol‐chloroform extraction method followed by PCR targeting *oxa-Ab* [15, 16].

### Antibiotic susceptibility testing

The antibiotics susceptibility of isolates was determined using the disk diffusion method according to CLSI guidelines [17] and against the following antibiotics: imipenem (10 mg), ceftazidime (30 mg), ceftriaxone (30 mg), amikacin (30 mg), gentamicin (10 mg), tetracycline (30 mg), piperacillin/tazobactam (110 mg), ampicillin/sulbactam (20 mg), ciprofloxacin (5 mg), levofloxacin (5 mg) and trimethoprim/sulfamethoxazole (25 mg) (Mast Group Ltd, UK). The CRAB was defined when the isolate was resistant to imipenem [16].

### Detection of OXA-type carbapenemases genes

A multiplex PCR was performed to detect *oxa23*, *oxa24* and *oxa58* genes in *A. baumannii* isolates as previously described [18].

### Detection of *bla*_TEM_ and *bla*_NDM-1_

PCRs were performed to detect *bla*_TEM_ and *bla*_NDM-1_ genes in *A. baumannii* isolates as previously described [19, 20].

### Identification of global clones

Two multiplex PCR assays amplifying alleles of *ompA*, *csuE* and *oxa-Ab* were performed to identify group 1 (GC2) or group 2 (GC1) as previously described [15].

### Multi-locus sequence typing (MLST)

Genetic relatedness of isolates was determined using the Institut Pasteur (IP) multilocus sequence typing scheme. Based on the IP scheme, seven housekeeping genes including *cpn60*, *fusA*, *gltA*, *pyrG*, *recA*, *rplB*, and *rpoB* were amplified and subjected to sequencing. A sequence type (ST) number was assigned to each isolate according to the allele sequences available on the MLST site (http://pubmlst.org/abaumannii/) [21].

### Statistical analysis

Data analysis was performed using SPSS version 18.0 (SPSS Inc., USA). Descriptive results were shown as frequencies and mean. For comparison of the categorical variables, Chi-square and Fisher’s exact tests of nonparametric data were used.

## Results

### Characteristics of COVID-19 patients

Eighteen (7.4%) out of 242 COVID-19 patients were diagnosed with CRAB co-infection (mean age of 63.94 ± 13.8 years, 12 male and 6 female), of whom 14 were suffering from underlying diseases, such as asthma, cardiovascular diseases, lymphoma, hypothyroidism, diabetes, necrotizing fasciitis, hypertension or chronic kidney disease (Table 1). The mean LOS in ICU for co-infected patients was 13.55 ± 9.15 days and the majority of them (16 of 18) required mechanical ventilation and intubation. Despite different antimicrobial treatments shown in Table 1, death was recorded as the outcome in ICU for 9 (50%) COVID-19 patients co-infected with CRAB.

**Table 1:** Properties of carbapenem-resistant *A. baumannii* (CRAB) isolates recovered from COVID-19 patients admitted to an Iranian referral hospital at the first wave of COVID-19

COVID-19 patient	Date	Gender	Age (years)	Ward	Length of stay at ICU (days)	Mechanical ventilation	Intubation	Underlying disease	Site	Antibiotic treatment	Outcome	Isolate	*oxa* genes^a^	*bla*_TEM_	*bla*_NDM-1_	ST
Patient 1	Apr 1, 2020	Male	75	ICU1	1	Yes	Yes	No	Blood	Meropenem, amikacin, ciprofloxacin, linezolid and sulfamethoxazole	Death	AB7	23 and 24	+	−	2
Patient 2	Apr1, 2020	Male	67	ICU2	12	Yes	Yes	Cardiovascular disease	Blood	Meropenem and colistin	Survive	AB8	23	−	−	2
Patient 3	Apr4, 2020	Male	85	ICU3	31	Yes	Yes	Hypertension, cardiovascular disease, tracheostomy	Tracheal discharge	Imipenem	Death	AB12	23	−	−	2
Patient 4	Apr3, 2020	Male	46	ICU3	4	No	No	Hypertension and chronic neuromuscular diseases	Blood	Vancomycin,	Survive	AB14	23 and 24	+	−	2
Patient 5	Apr3, 2020	Male	57	ICU4	28	Yes	Yes	Asthma	Blood	Imipenem and linezolid	Survive	AB16	23 and 24	+	−	2
Patient 6	Apr 4,2020	Male	60	ICU5	15	Yes	Yes	Hypertension, cardiovascular disease and mitral valve replacement	Blood	Meropenem	Death	AB18	23	−	−	2
Patient 7	Apr 8,2020	Female	48	ICU2	11	Yes	Yes	Lymphoma and history of chemotherapy	Tracheal discharge	Levofloxacin and azithromycin	Survive	AB25	23	−	−	2
Patient 8	Apr 8, 2020	Female	63	ICU5	29	Yes	Yes	Osteoporosis and tracheostomy	Tracheal discharge	Imipenem, colistin, ampicillin/sulbactam and piperacillin/tazobactam	Survive	AB26	23 and 24	−	−	2
Patient 9	Apr 10, 2020	Male	62	ICU2	4	Yes	Yes	Asthma, cardiovascular diseases and respiratory disease	Tracheal discharge	Azithromycin	Death	AB27	23 and 24	−	−	2
Patient 10	Apr 12, 2020	Male	39	ICU3	18	Yes	Yes	No	Tracheal discharge	Meropenem, colistin and vancomycin	Death	AB29	23	−	−	2
Patient 11	Apr 13, 2020	Male	87	ICU5	17	Yes	Yes	Asthma	Tracheal discharge	Piperacillin/tazobactam, vancomycin, meropenem, colistin, amikacin, gentamicin, ciprofloxacin, sulfamethoxazole, ceftriaxone, ceftazidim	Death	AB35	23	−	−	2
Patient 12	Apr 15, 2020	Female	53	ICU3	8	Yes	Yes	No	Tracheal discharge	No	Death	AB36	23	−	−	2
Patient 13	Apr 16, 2020	Female	73	ICU5	6	Yes	Yes	Hypothyroidism	Tracheal discharge	Vancomycin, linezloid, meropenem, colistin and levofloxacin	Survive	AB39	23	−	−	2
Patient 14	Apr 16, 2020	Female	71	ICU5	22	Yes	Yes	Asthma and diabetes	Blood	Colistin, gentamicin and sulfamethoxazole	Death	AB40	23	−	−	2
Patient 15	Apr 18, 2020	Male	66	ICU4	10	No	No	Necrotizing fasciitis	Tracheal discharge	Meropenem, ciprofloxacin, tazocin, imipenem, sulfamethoxazole	Survive	AB41	23	−	−	2
Patient 16	Apr 18, 2020	Male	74	ICU5	11	Yes	Yes	Diabetes	Blood	Meropenem, colistin, piperacillin/tazobactam and gentamicin	Death	AB44	23	−	−	2
Patient 17	Apr 25, 2020	Female	46	ICU3	14	Yes	Yes	No	Tracheal discharge	Colistin	Death	AB55	23	−	−	2
Patient 18	Apr 26, 2020	Male	79	ICU2	3	Yes	Yes	Chronic kidney disease	Tracheal discharge	Imipenem,meropnem, colistin	Survive	AB56	23	−	−	2

^a^Numbers indicate the carbapenemase resistance genes; *oxa23*, *oxa24* and *oxa58*

### Properties of the CARB isolates

From 18 CRAB isolates, 11 (61.2%) isolates were recovered from tracheal discharge and the remaining 7 (38.8%) isolates were obtained from blood. The CRAB isolates had similar resistance profiles in the susceptibility test against ceftriaxone amikacin, gentamicin, ampicillin/sulbactam, piperacillin/tazobactam and ciprofloxacin. All CRAB isolates carried *oxa23*, whereas 5 of them harboured *oxa24* (Table 1). All CRAB isolates belonged to GC2 and were found to contain the same MLST profile of 2-2-2-2-2-2-2, which corresponds to ST2_IP_. These isolates were obtained from COVID-19 patients who were admitted to different wards as follows: ICU1 (n = 1), ICU2 (n = 4), ICU3 (n = 5), ICU4 (n = 2) and ICU5 (n = 6). In the ICUs in which more than one CRAB isolates were found, the number of the isolates containing only *oxa23* was higher than those carrying both *oxa23* and *oxa24*. Three isolates contained *bla*_TEM_ and no isolates carried *bla*_NDM-1_.

## Discussion

CRAB is one of the emerging causes of HAI, particularly among patients who require devices such as ventilators and blood catheters [1]. In 2017, CRAB was grouped by World Health Organization (WHO) in the list of critical (priority 1) pathogens according to the urgent need for research and development on novel antimicrobial agents [22]. This bacterium causes untreatable infections due its high level of antibiotic resistance and as well as its arsenal of virulence factors, responsible for high mortality rates [23, 24]. Of major concern is that CRAB has the potential for transmission of resistance determinants via mobile genetic elements among the strains in healthcare settings [7]. Therefore, the timely recognition of CRAB, especially in the severely ill patients, is of prime importance. Here, we screened for CRAB among COVID-19 patients who were admitted to different wards in a hospital and investigated the clonal relatedness of the CRAB isolates.

This study showed that one-tenth of COVID-19 patients were co-infected with CRAB, regardless of the need for mechanical ventilation or intubation and of the LOS in ICU. Despite receiving monotherapy or combination therapy, death occurred in a half of patients who had the co-infection of SARS-CoV-2 and CRAB. Another study conducted in Qom, Iran, reported the bacterial co-infection in all COVID-19 patients (n = 19); of which, 90% died after enduring co-infection of multi-drug resistant *A. baumannii* and SARS-CoV-2 [25]. This rate of co-infection is significantly higher than what we found in the current study. There are also two studies from France [26] and South Africa [27], each reported one case of co-infection of *A. buamannii* and SARS-CoV-2 with an underlying disease. These studies, however, did not present the resistance profile of the *A. baumannii* isolate recovered from COVID-19 patients [26, 27]. Ramadan et al., reported the isolation of *A. baumannii* in 7 out of 260 patients with COVID-19 in Egypt and showed that 5 and 3 isolates were resistant to imipenem and meropenem respectively [28]. The limited therapeutic options against *A. buamannii* and SARS-CoV-2 highlight the importance of clinical management of complicated cases of COVID-19 when co-infected with antibiotic resistant *A. baumannii* [14]. The resistance profile of the *A. baumannii* isolates from COVID-19 patients was previously reported in a study by Sharifipour et al.; however, using phenotypic methods [25], it was unable to explain the possible mechanisms conferring resistance to carbapenems as the front-line treatment for antibiotic-resistant bacteria.

This study revealed that *oxa23* was linked to the carbapenem resistance as all CRAB isolates carried this gene. The *oxa24* gene also is associated with the carbapenem resistance in some isolates tested here. Although several studies show that *oxa23* is the most prevalent gene conferring resistance to carbapenem worldwide [29, 30], it mainly enters into chromosomes or plasmids via transposons [7] compared with *oxa24* which is commonly found to be located on plasmids [31–33]. As a limitation, this study did not determine the genetic contexts of *oxa23* and *oxa24* to show whether they were implicated in the dissemination of carbapenemase genes among different isolates in the hospital. Ramadan et al., detected the resistance genes other than OXA genes, including NDM-1, TEM and CTX-M in *A. baumannii* isolated from COVID-19 patients [28]. We found TEM in three isolates and NDM-1 in none of the isolates.

The assessment of clonal relatedness of the CRAB isolates recovered form COVID-19 patients in different wards at a hospital in Tehran showed that the CRAB isolates belonged to GC2 and the same sequence type (ST2 or ST2_IP_ according to Institut Pasteur scheme). Based on the available genome sequences, ST2 is the most frequent sequence type globally [7]. Previous studies in non-COVID-19 patients showed that the *A. baumannii* belonging to ST2 of GC2 caused outbreaks in hospital settings [34–36]. As yet, there is no study showing the clonal distribution of *A. baumannii* isolated from COVID-19 patients. Although the CRAB isolates examined here were obtained from patients in different ICUs, they shared *oxa23*, the sequence type and the phenotypic resistance to several antibiotics. However, a subset of isolates was found to harbour *oxa24*. This finding may explain the possible colonization of the hospital which is either with the CRAB belonged to ST2 or with the CRAB entered the hospital at some time and subsequently undergone the genetic events resulting in the emergence of the isolates carrying *oxa24*. This hypothesis needs to be confirmed by metagenomic analysis of the isolates in future studies.

Information on the circulation of CRAB in different ICUs of the hospital before the pandemic revealed the existence of 25 CRAB isolates within 6 months (September 2019 to February 2020); of which, 22 belonged to GC2 and the remaining 3 isolates were non-typeable using multiplex PCRs for identifying the global clone (data not shown). Considering the number (n = 25) of CRAB isolates within 6 months, recovery of 18 isolates during two months in the first wave of the pandemic is unexpected and may imply an outbreak.

## Conclusion

This study provided evidence that CRAB isolates belonging to ST2_IP_ and sharing the *oxa23* gene have caused several outbreaks in COVID-19 patients. Although the main characteristics of these isolates represented their clonal relatedness, they differ in the antibiotic resistance genes which are mainly encoded on plasmids.

## Data Availability

All data generated or analysed during this study are included in this published article.

## Abbreviations

*bla*: β-Lactamase
CTX-M: Cefotaximase-Munich
CDC: Centers for Disease Control and Prevention
CRAB: Carbapenem-resistant *Acinetobacter baumannii*
FDA: U.S. Food and Drug Administration
GC: Global clones
HAI: Health care-associated infections
IPC: Infection and prevention control
IP: Institut Pasteur (IP)
MLST: Multi-locus sequence typing
ICU: Intensive care unit
LOS: Length of stay
*oxa*: Oxacillinases
ST: Sequence type
SARS-CoV-2: Severe acute respiratory syndrome coronavirus 2
RT-PCR: Reverse transcription-polymerase chain reaction
VAP: Ventilator-associated pneumonia
